# Role of Potash Alum in Hepatitis C virus Transmission at Barber's Shop

**DOI:** 10.1186/1743-422X-8-211

**Published:** 2011-05-09

**Authors:** Yasir Waheed, Sher Zaman Safi, Ishtiaq Qadri

**Affiliations:** 1 NUST Center of Virology and Immunology, National University of Sciences and Technology, H-12 Sector, Islamabad, Pakistan

## Abstract

Hepatitis C virus (HCV) is the main cause of severe liver disease, including hepatocellular carcinoma, cirrhosis and end stage liver disease. In Pakistan most of HCV positive patients have history of facial/armpit shaving from barbers. 79% of barbers are rubbing Potash Alum stone on facial shaving cuts. Dark blood spots are analyzed on Potash Alum stones being used at different barber shops. The aim of the study was to check the viability of hepatitis C virus on potash alum stone being used at barber shops. Blood samples from HCV positive patients were taken and treated with 0.1, 0.2, 0.3, 0.4 and 0.5 molar concentrations of Potash Alum for different periods of time. Blood was centrifuged to isolate the serum; HCV RNA was extracted from serum and subjected to first strand synthesis and PCR. PCR fragments were confirmed by sequencing. PCR amplification was observed in all the samples, treated with different concentrations of Potash Alum, indicated that the virus remains alive on Potash Alum stone for a long period of time. Potash Alum being used by barbers on facial shaving cuts has definite role in HCV transmission in Pakistani population. Therefore use of Potash Alum stone should be banned on facial shaving cuts at barber shops.

## Introduction

Hepatitis C virus is the main cause of severe liver disease, including hepatocellular carcinoma, cirrhosis and end stage liver disease [1]. About 200 million people are infected with this virus which covers about 3.3% of world's population [2,3]. HCV infection leads to chronic infection in 50% to 80% of individuals [4]. It was estimated by world health organization in 2004 that the annual deaths due to liver cancer caused by HCV and cirrhosis were 308,000 and 785,000 respectively [5]. In Pakistan the prevalence of HCV is 5% in general population [6].

Health has been considerd as fundamental human right [7] and is a key refractor of distribution of resources in a society [8]. Pakistan is a developing country of 180 million people with low health and educational standards. It was ranked 134^th ^out of 170 countries according to the human development index of United Nations [9]. In developing countries due to non implementation of international standards regarding blood transfusion, shaving from barbers, reuse of needles for ear and nose piercing, reuse of injections, injecting drug users, tattooing, unsterilized dental and surgical instruments are the main source of HCV transmission [10].

The word barber originates from Latin word Barba meaning beard. Barber is a person whose occupation is to cut any type of hair, trimmed beard and give shave [11]. There is strong evidence that razors, barber's scissors, nail files and body piercing instruments are risk factors for transmitting hepatitis C. It is reported that hepatitis B can survive outside the body for seven day or more on table tops, workbenches and other instruments [12]. Barbers are also involved in incision, circumcision and drainage of abscesses especially in rural areas and only 13% of them know that hepatitis C is a disease of liver, causing cancer [13].

Potash alum or potassium alum is potassium double sulfate of aluminum. It is an astringent, styptic and antiseptic. It also has cosmetic uses as a deodorant and as an aftershave treatment. In ancient Babylon, physicians used alum in a mouthwash, as a pessary for menorrhagia, as treatment for itchy scabs, gonorrhea and purulent ophthalmia. For this reason, it can be used as a natural deodorant by inhibiting the growth of the bacteria responsible for body odor. Its astringent and styptic properties are often employed as after shaving and to reduce bleeding in minor cuts and abrasions [14,15]. It has been reported in our previous study that 79% of barbers are using potash alum stone on facial cuts and as after-shave. They are rubbing same stone on more than hundred shaves and facial cuts, dark blood spots are seen on the potash alum stone being used at different barber's shops. It may be a factor in transmission of hepatitis C in Pakistani population [16].

In order to evaluate the antiviral effect of potash alum and viability of virus particle on potash alum, blood samples of HCV positive patients were taken and treated with various concentrations of potash alum for different duration of time, nucleic acid was extracted and then subjected to PCR. Viability of virus particles was analyzed with the intensity of PCR bands.

## Materials and methods

### Sample collection and Treatment

Serum samples of HCV positive patients were taken from the diagnostic department of NUST Center of Virology and Immunology. 200 ul of serum samples were taken and treated with 200 ul of 0.1, 0.2, 0.3, 0.4 and 0.5 molar concentration of potash alum solution for six hours. The mix of serum plus potash alum becomes semi solid mass due to high molar mass of the salt. 300 ul of nuclease free water was added, vortexed vigorouly and then centrifuged at 12000 × g for 2 minutes to get the treated serum.

### RNA extraction and RT-PCR

Serum collected from the centrifugation was subjected to RNA extraction by using the Qiagen (Germany) RNA extraction kit according to the manufacturer's protocol. Primers were designed from the sequence comparison of HCV NS5B regions with accession numbers of NC009824, AM423015, AM423018, AM423014, AM423017, D17763, D28917 by using CLC workbench software. The sequence of the primers were 5'-ATCTCCTCCCTCACGGAGCG-3'

5'-CGATCAAGTATCTCCTGGGATTGGA-3'.

The RNA extracted was taken as template for the Complementary DNA (cDNA) synthesis. The reaction mixture for reverse transcription had a total volume of 20 ul contained 13 ul of RNA, 1 ul dNTPs (10 mM), 4 ul M.Mulv Buffer, 1 ul M.Mulv enzyme and 1 ul specific antisense primer. Cycle conditions for cDNA were as follows: 42°C for 55 min followed by 70°C for 10 minutes.

The PCR reaction conditions were as follows: reaction mixture contained 5 ul of cDNA as template, 1 ul of each sense and antisense primer, 2 ul of dNTPs (2 mM), 2.5 ul of Dream Taq buffer, 13 ul of nuclease free water and 1.5 unit of DreamTaq Enzyme (Fermentas). The cycle conditions were as follows: 94°C for 3 minutes followed by 35 cycles of 94°C for 45 seconds, 62°C for 45 seconds, 72°C for 60 seconds and a final extension at 72°C for 7 minutes. Amplified PCR products were analyzed by electrophoresis on 1.2% agarose gel containing ethidium bromide. PCR band confirmation was done by sequencing of fragments.

## Results and Discussion

Serum of HCV positive patients were taken and treated with different concentrations of potash alum for long periods of time. The mix of serum plus potash alum becomes semi solid mass due to high molar mass of the salt. 300 ul of nuclease free water was added, vortexed vigorouly and then centrifuged at 12000 × g for 2 minutes to get the treated serum. Ribonucleic Acid was extracted from serum and then subjected to first strand DNA synthesis. Specific primers were designed from the conserved region of the NS5B of HCV. These primers amplify a specific 565 base pairs band in PCR reaction.

**Figure 1:**
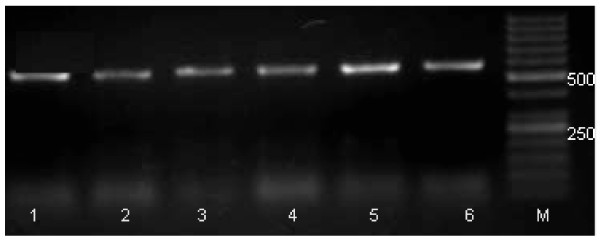
**Lane 1 to 6 contains PCR bands of 580 base pairs**. Lane 1 contains untreated sample, Lane 2-6 contains serum samples treated with 0.1, 0.2, 0.3, 0.4 and 0.5 molar concentration of potash alum. M is the 50 base pairs marker.

Treatment of serum samples with potash alum has no antiviral effect, as PCR amplification takes place in all the treated samples. There was no significant change in PCR band intensity in serum samples treated with various concentrations of Potash Alum. Specific PCR bands of 565 bp were analyzed on agrose gel, as shown in Figure 1.

The present study was conducted to check the viability of hepatitis C virus (HCV) in the presence of potash alum stone and to predict its role in transmission of HCV in Pakistani population. Barbers in third world countries are mostly unaware of transmission of infectious agents by the repeated use of scissors and razors on multiple clients without sterilization. The prevalence of road side barber shaving has been reported very high (34% - 49%) in countries like Bangladesh, Ethiopia and Pakistan. In Africa barber shaving is one of the major non-sexual cultural practice which may expose the individuals to blood and blood borne pathogens through the use of shared instruments, ritual scarification, genital tattooing and group circumcision [17]. HCV prevalence is high in Japanese institutionalized psychiatric patients due to the use of same razor on multiple clients [18].

**Figure 2:**
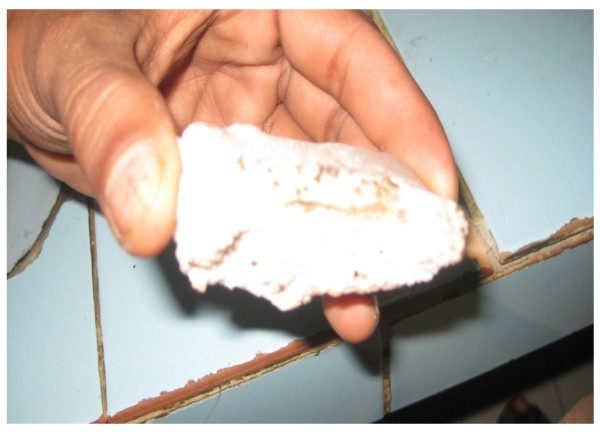
**Dark blood spots are visible on potash alum being used at barbers shop**.

It was reported in a survey conducted at capital twin cities of Pakistan, in which 508 different barber shops were visited that 79% of barbers were rubbing potash alum stone on the facial cuts or as after shave. Some barbers had huge work load, doing more than hundred shaves per day and applying same stone on multiple clients increased the chances of pathogen transmission from this source. Potash alum was used as antiseptic and dark blood spots remained visible on the stone (Figure 2) and then applying same stone on the next client increased the chance of transmission of pathogens [16].

Potash alum has high molar mass of 258 g/mol and it's hard to make one molar solution of it. 0.1, 0.2, 0.3, 0.4 and 0.5 molar solution of Potash Alum was made and serum samples of HCV positive patients were treated for six hours. The mix of serum and potash alum becomes a solid mass. PCR grade water was added, vortexed vigorously to get the serum and that serum was proceed to amplification of HCV specific regions. Same intensity of PCR band was observed in all the treated samples, showing that potash alum does not have any antiviral effect. Hepatitis C virus remains alive in the presence of potash alum for several hours. As dark blood spots were seen on different potash alum stones being used at barber's shop, it is definite factor in the transmission of hepatitis C virus in Pakistani population. It should be banned to use potash alum stone on facial shaving cuts or as after shave.

In Asia and Africa, HIV and hepatitis control and prevention programs are increasingly recognizing the importance of providing infection control and education for healthcare professionals. Such education should focus on the importance of proper sterilization techniques, avoidance of re-use and sharing of contaminated equipment and supplies. Population based efforts including educational activities for barbers, on safe grooming practices are required that can minimize the spread of these deadly blood borne viruses [17].

## Conclusion

Barbers are applying potash alum stone at facial shaving cuts. When they apply potash alum stone on HCV positive patient, the infected blood attaches on the stone. The virus remain infectious on the stone for a long period of time and applying same stone on normal person's shaving cuts is the definite route of transmission of HCV. So it should be banned to apply potash alum stone on facial shaving cuts at barbers shop.

## Competing interests

The authors declare that they have no competing interests.

## Authors' contributions

YW collected samples, treated with potash alum, extracted nucleic acids, perform PCR and wrote manuscript. SZS help YW in extraction and analysis. IQ supervised all the work. All the authors read and approved the final manuscript.
